# Comparative hydrolysis analysis of cellulose samples and aspects of its application in conservation science

**DOI:** 10.1007/s10570-021-04048-6

**Published:** 2021-07-23

**Authors:** Manuel Becker, Kyujin Ahn, Markus Bacher, Chunlin Xu, Anna Sundberg, Stefan Willför, Thomas Rosenau, Antje Potthast

**Affiliations:** 1 Department of Chemistry, Institute of Chemistry of Renewables, University of Natural Resources and Life Sciences, Muthgasse 18, Vienna, 1190 Austria; 2 c/o Laboratory of Natural Materials Technology, Johan Gadolin Process Chemistry Centre, Åbo Akademi University, Porthaninkatu 3, 20500 Turku, Finland; 3 National Archives of Korea, 30 Daewangpangyo-ro 851beon-gil, Sujeong-gu, Seongnam-si, Korea

**Keywords:** Cellulose, Crystallinity, Electron beam irradiation, Hemicellulose, Methanolysis, Sulfuric acid hydrolysis, Historic paper

## Abstract

Knowledge about the carbohydrate composition of pulp and paper samples is essential for their characterization, further processing, and understanding the properties. In this study, we compare sulfuric acid hydrolysis and acidic methanolysis, followed by GC–MS analysis of the corresponding products, by means of 42 cellulose and polysaccharide samples. Results are discussed and compared to solid-state NMR (crystallinity) and gel permeation chromatography (weight-averaged molecular mass) data. The use of the hydrolysis methods in the context of cellulose conservation science is evaluated, using e-beam treated and artificially aged cellulose samples.

**Supplementary Information:**

The online version contains supplementary material available at 10.1007/s10570-021-04048-6.

## Introduction

The carbohydrate composition of pulp and paper samples is a key parameter for the characterization of their chemical and physical properties and for their further processing (Willför et al. 2005a, b, 2009; Sundberg et al. 2003). While chemically pure cellulose is theoretically only composed of (anhydro)glucose units, real-world cellulosic samples are far from being that ideal: pulps contain hemicelluloses, papers contains sizing agents and fillers, e.g. starch, and historic paper samples may contain stabilizers and additives. Gum Arabic or pectins are two well-known and analytically well characterized examples, but often those additives are of unknown structure and origin. Obtaining knowledge about the carbohydrate composition is obviously a first and important step in the material characterization. In most cases, carbohydrate composition is determined by hydrolysis and quantification of the obtained monomers by chromatographic methods (Black and Fox 1996). Alternative spectroscopic methods on the intact polymer, such as IR or NMR, are insufficient when it comes to quantification of the contained monosaccharides, especially of minor components. Hydrolysis, in this regard, is synonymous with cleavage of the glycosidic bonds of the polysaccharides. Both enzymatic and acidic hydrolysis, when performed exhaustively, release the monosaccharides as the final subunits of a polysaccharide. Acid hydrolysis can involve sulfuric acid, trifluoroacetic acid, or hydrochloric acid under strong conditions, mostly in aqueous medium, or milder environments, e.g. in organic solvents with catalytic amounts of acid (Bertaud et al. 2002). The liberated monosaccharides are usually subsequently analyzed by HPLC (Bose et al. 2009; Galant et al. 2015), GC (Sundberg et al. 1996) or NMR (Marques et al. 2010; Duquesnoy et al. 2008).

There are obvious differences in the rates of acidic hydrolysis of poly- and oligosaccharides which originate in reactivity differences of the glycosidic linkages. The type of monosaccharide (e.g. glucose vs. mannose vs. xylose), anomer (α/β), linkage (e.g. β-*O*-3 vs. β-*O*-4, vs. β-*O*-6), neighboring activating or deactivating groups (such as ether, keto or carboxylic acid moieties) and their position, and the surrounding hydrogen bond system are major influencing factors (Yoneda et al. 2016). Hydrolysis efficiency is of course also affected by the conditions, such as the type, strength and concentration of acid used, duration of the hydrolysis treatment, and reaction temperature (Panagiotopouos 2005). Two general hydrolysis approaches, with several sub-variants, have become the generally accepted and frequently used standard approaches for compositional carbohydrate analysis: total hydrolysis with sulfuric acid and acidic methanolysis. Each of it has its own advantages and disadvantages which have to be weighed against each other for a particular application or sample set.

Hydrolysis with sulfuric acid converts polysaccharides into monosaccharides. The advantage of the method is the high conversion, reflected in mostly complete solubilization of the starting material and the common term “total hydrolysis”. Also substrates that are “hydrolysis-resistant” because of physical traits (high crystallinity) or attenuating chemical influences (polyuronic acids) are reliably converted. However, the strong acidity and the harsh conditions entail lots of side reactions as a major drawback. Uronic acids decarboxylate, sulfuric acid groups might be introduced, and monosaccharides once formed, in particular keto sugards, are converted into their primary furanoid condensation/dehydratization products, such as furfural or 5-hydroxymethylfurfural, and follow-up compounds.

Relative to sulfuric acid-based total hydrolysis, acidic methanolysis represents a comparatively mild hydrolysis method, which largely avoids such side reactions, albeit at the expense of incomplete conversion of hard-to-hydrolyze material (Chambers and Clamp 1971). The liberated monosaccharides are converted into their corresponding methyl glycosides, and carboxyl units of uronic acids are converted into their methyl esters (Huang et al. 1992). The methyl glycosides formed lose their anomeric information and equilibrate to α- and ß-furanoses and pyranoses (Laine et al. 2002). The fact of increased analytical complexity—a single monosaccharide can appear in the form of up to four cyclic isomers after acidic methanolysis—was often portrayed as severe drawback, but is now viewed in a more differentiated way. Evidently, the formation of more isomers per monosaccharide leads to an increase in chromatogram complexity and may decrease the sensitivity of analysis because signal intensity of one analyte is distributed over several peaks (Rumpel and Dignac 2006). However, this signal splitting reduces the frequent risk of complete peak overlapping, and the constant ratio of isomers for a given monosaccharide enables compound identification and quantification on one of the up to four peaks (Laine et al. 2002; Amelung et al. 1996). The advantage of acidic methanolysis, compared to sulfuric acid hydrolysis, is much less unwanted degradation and byproduct formation, in particular of fragile hemicelluloses, and the possibility to assess and quantify uronic acids (Chambers and Clamp 1971). This is offset by the fact that crystalline cellulose domains are affected only slightly, so that for such material the strong acid hydrolysis methods is recommended (Sundberg et al. 1996). Unfortunately, a hydrolysis method that combines the best of both worlds—complete hydrolysis of the resistant lignocellulose and no side reactions of the labile hemicellulose degradation products—does not yet exist. Thus, total glucose, pentoses, and uronic acids are not accessible by a single hydrolysis method, so both approaches must still be combined to get maximum information.

The carbohydrate composition of papers and similar cellulosic materials shows a predominance of glucose, evidently from cellulose being the major constituent, and smaller amounts of pentoses, hexoses, or deoxy sugars and sugar acids. These originate from the hemicellulose fraction (glucuronoxylan, galactoglucomannan) or from carbohydrate-based stabilizers or additives (Gum Arabic, guar gum, pectins).

In this study, the mentioned two hydrolysis methods, sulfuric acid hydrolysis and acidic methanolysis, were applied to characterize monomer composition of cellulose samples and related polysaccharides, covering a wide range of monosaccharides and sugar acid compounds. The determination of the whole cellulose/total glucose content was carried out by sulfuric acid hydrolysis according to Bose et al. (2009) The analysis of the hemicellulose fractions, pectins and additives adopted the method of Sundberg et al. (1996), using acidic methanolysis followed by derivatization/gas chromatography (GC). Attempts were made to determine whether the methods would be useful in characterizing the effects of electron beam irradiation (e-beam) or cellulose conservation treatments such as artificial aging, and whether they would indicate changes in monomer composition upon such treatments. Electron beam radiation can be used to treat mold infested collections to deactivate microorganisms. The hydrolysis data from the two alternative methods were compared and correlated with data from solid-state NMR spectroscopy (crystallinity) and gel permeation chromatography analysis (weight-average molar mass, M_w_).

## Material and methods

### Chemicals and reagents

The reference compounds D-(-)-arabinose, D-(+)-galactose, D-(+)-glucose, D-(+)-mannose, L-(+)-rhamnose (6-deoxy-mannose), D-(+)-xylose, D-(+)-galacturonic acid monohydrate (GalA), D-glucuronic acid (GlcA), the internal standard sorbitol, anhydrous pyridine, acetic acid, ethyl acetate, sodium carbonate (Na_2_CO_3_), *N*,*O*-bis(trimethylsilyl)trifluoroacetamide (BSTFA), trimethylchlorosilane (TMCS) and 4-(dimethylamino)pyridine (DMAP) were purchased from Sigma-Aldrich/Fluka (Sigma-Aldrich Schnelldorf, Germany). All standards, chemicals, and reagents were of p.a. grade and used without further purification.

### Materials

All polysaccharide (Table 1), cellulose pulp (Table 2) and paper (Table 3) samples were freeze-dried prior analysis. For acidic methanolysis, amounts of 1–2 mg in the case of polysaccharides and 10 mg (± 2 mg) in the cellulose pulps or papers were used for analysis. The sulfuric acid hydrolysis was conducted with sample amounts of 40 mg (± 1 mg) for all substrates.

**Table 1:** Sample list of polysaccharides analyzed

	Polysaccharides	Code	Origin	Producer
P1	Arabinan	AS	Sugar Beet	Megazyme
P2	Arabinan—Debranched	DA	Sugar Beet	Megazyme
P3	Arabinan—Linear 1,5-α-L	L < A	Sugar Beet	Megazyme
P4	Galactan	GG	Potato	Megazyme
P5	Pectic galactan	GL	Lupin	Megazyme
P6	Pectic galactan	GP	Potato	Megazyme
P7	Galactomannan	GC	Carob	Megazyme
P8	Galactomannan	GB	Locust bean	Sigma
P9	Glucogalactomannan	GS	Spruce	Åbo Akademi University
P10	Glucomannan	GK	Konjac	Megazyme
P11	Gum arabic	GA	Acacia tree	Sigma-Aldrich
P12	Inulin	IN	Dahlia tubers	Sigma-Aldrich
P13	Pectin Classic AU202	AU	Apple	Herbstreith and Fox KG
P14	Pectin Classic CM201	CM	Citrus	Herbstreith and Fox KG
P15	Pectin, esterified	PC	Citrus	Sigma
P16	Polygalacturonic acid	PG	Orange	Sigma
P17	Rhamnogalacturonan	RG	Soy Bean	Megazyme
P18	Stachyose	ST	Stachys tuberifera	Sigma-Aldrich
P19	Xylan	LG	Beech	Lenzing AG
P20	Xylan	XB	Birch	Sigma
P21	Xyloglucan	XG	Tamarind	Megazyme

**Table 2:** Sample list of cellulose (pulp) samples analyzed

	Sample	Code	Origin	Producer
F01	Cotton Linters	CL	Cotton	Buckeye
F02	Wheat bran	BR	Wheat	Unknown
*Hardwood pulp*				
F03	Bleached Hardwood-Kraft pulp	HK	Birch	Unknown
F04	Bleached Hardwood-Sulfite pulp	HS	Beech	Lenzing AG
*Softwood pulp*				
F05	Bleached Softwood-Kraft pulp	SK	Spruce (70%), pine (30%)	Södra
F06	Bleached Softwood-Sulfite pulp	SS	Spruce	Domsjö
F07	Bleached Softwood-TMP	TM	Spruce	Åbo Akademi University
F08	Bleached Eucalyptus paper pulp—Kraft pulp	EC	Eucalyptus	ENCE
F09	Bleached Eucalyptus paper pulp—Kraft pulp e-beam treated*	EC-E	Eucalyptus	ENCE
F10	Bleached Hemp paper pulp ECF	HC	Hemp	Celesa
F11	Bleached Hemp paper pulp ECF—e-beam treated*	HC-E	Hemp	Celesa

*Beta-irradiation of 120 kGy

**Table 3:** Sample list of papers analyzed

No	Sample	Code	Origin
F12	Book 1 (1951)	B1	
F13	Book 2 (1912)	B2	
F14	Book 3 (1892)	B3	
F15	Book 4 (1860)	B4	
F16	Mulberry paper	MB	Mulberry
F17	Paper sample (historical)	PH	
F18	Rag papr (historical)	RH	
F19	Rag paper (modern)	RM	
F20	Rag paper (modern)—artificially aged*	RM-A	
F21	Rag paper (modern)—e-beam treated**	RM-E	

*Accelerated aging conditions: 80 °C and 65% RH for two weeks

**Beta-irradiation of 60 kGy

### Sulfuric acid hydrolysis

Sulfuric acid hydrolysis of polysaccharides was conducted according to a procedure by Bose et al. (2009), which was modified employing a two-step treatment at different acid concentrations followed by derivatization-GC-MS analysis. In the primary hydrolysis step, 1.5 mL of 72% aqueous H_2_SO_4_ was added to the sample (40 ± 1 mg) in a vial, followed by stirring at room temperature for 2 h. For the second hydrolysis step, 2 mL of H_2_O was added and the mixture was heated in an oven at 80 °C for 1 h. The hydrolysis solution was cooled down in an ice bath and stored at 4 °C overnight. Internal standard solution (150 mg of sorbitol in 100 mL of H_2_O, 7 mL) were added to the hydrolysis solution. An aliquot of 1.5 mL was neutralized with solid Na_2_CO_3_ (approx. 290 mg) until bubble generation due to CO_2_ evolution subsided. The solution was filtered (0.45 µm, 13 mm diameter) into a new GC vial and the pH value was adjusted to 7 by adding 1–2 drops of acetic acid (control with indicator paper).

### Acidic methanolysis

The protocol was based on the procedure by Sundberg et al. (1996).^18^ In a vial, the dried sample materials (1–2 mg of polysaccharides or 10 ± 2 mg of cellulosic pulps/papers) were added into a solution of HCl in anhydrous methanol (2 M, 2 mL). A calibration solution (1 mL) containing 0.1 mg/mL of sugar monomers and uronic acids was subjected to acidic methanolysis in a separate vial but under the same conditions. The vials were sealed and the samples kept at 100 °C for 3 h. After cooling to room temperature, samples were neutralized by adding pyridine (100 µL). Internal standard solution (0.1 mg of sorbitol/mL methanol, 1 mL) was added to the samples (methanolysis sample and calibration mix), which were evaporated to dryness in a water bath (50 °C) under nitrogen until dryness and further dried in a vacuum desiccator at room temperature for 30 min.

### Per(trimethylsilylation) of hydrolysis product mixtures

The derivatization used the procedure by Becker et al. (2013a, b).^31^ The dried hydrolysates, calibration mixtures, and reference compounds were dissolved in 200 µL of pyridine and incubated at room temperature for 30 min. A solution of the silylation catalyst (1.5 mg/mL DMAP in pyridine, 200 µL) and silylation agent (*N,O*-bis(trimethylsilyl)trifluoroacetamide containing 10% trimethylsilyl chloride, 200 µL) was added to the mixture, which was stirred at 70 °C for 2 h. After cooling to r.t., the derivatized samples were kept at − 20 °C until analysis.

### GC-FID and GC–MS analysis of TMS-derivatized hydrolysis products

The derivatized samples were diluted with ethyl acetate (600 µl) and filtered before injection. Aliquots of 0.2 µL were injected in splitless mode and analyzed on an Agilent 7890A gas chromatograph coupled with an Agilent 5975C mass selective detector and Agilent GC Sampler 120. GC-FID analysis was performed on a Perkin Elmer Autosystem XL gas chromatograph with analysis parameters based on Sundberg et al. (1996).^18^ Column: HP-1 (25 m × 0.20 mm × 0.11 µm; J&W Scientific, Folsom, CA, USA); carrier gas: hydrogen, injector temperature: 250 °C; column flow: 0.8 ml/min, pressure 14 psi; oven program: 100 °C (1 min), 4 °C/min to 170 °C, 12 °C/min, 300 °C (7 min); detector temperature: 310 °C. Aliquots of 1 µL were injected in split mode (split ratio 1:25).

General GC–MS analysis conditions: Column: HP-5MS (30 m × 0.25 mm × 25 µm; J&W Scientific, Folsom, CA, USA); carrier gas: helium, MS: EI mode, 70 eV, source pressure: 1.13 × 10^−7^ Pa, purge flow: 36.3 ml/min, 0.6 min; source temperature: 230 °C. Scan range was set from 43 to 950 Da. Parameters for analysis of products from acidic methanolysis: injector temperature: 140 °C (30 °C/min to 260 °C); column flow: 0.9 ml/min; oven program: 140 °C (1 min), 4 °C/min to 210 °C, then 30 °C/min, 260 °C (5 min); inlet pressure 78.361 kPa.

Parameters for analysis of products from sulfuric acid hydrolysis: injector temperature: 150 °C (30 °C/min to 260 °C); column flow: 0.9 ml/min; oven program: 120 °C (2 min), 5 °C/min to 230 °C, then 20 °C/min, 260 °C (10 min); inlet pressure 78.361 kPa.

### Peak identification and quantification

**Fig. 1:**
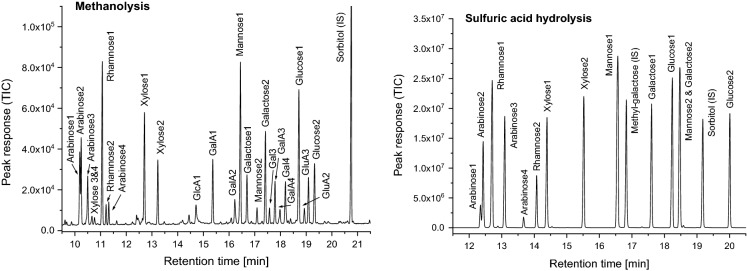
Left: acidic methanolysis of a carbohydrate mixture containing arabinose, rhamnose, xylose, galactose, glucose, mannose, galacturonic acid (GalA), and glucuronic acid (GlcA); right: sulfuric acid hydrolysis of a carbohydrate mixture containing arabinose, rhamnose, xylose, galactose, glucose, and mannose

Peak assignment, data acquisition, and quantification of hydrolysis or methanolysis products were performed with MSD Chemstation E.2.01.1177 (Agilent Technologies, USA). Peaks were assigned by comparing their retention times and mass spectra with those of corresponding reference compounds (Fig. 1). Calibration factors were determined from the carbohydrate standard solution after sulfuric acid hydrolysis or acidic methanolysis by the ratio between the total area of the different peaks of one analyte and the area of the sorbitol peak. The calibration factor of 4-*O*-MeGlcA, which is not commercially available as pure standard, was approximated to be the same as for GlcA. Samples were analyzed in quadruplicate, and values deviating from the average by more than 15% were regarded as outliers. All the results were based on masses of dried and freeze-dried material.

### Solid-state NMR

All solid-state NMR experiments were performed on a Bruker Avance III HD 400 spectrometer (resonance frequency of ^1^H of 400.13 MHz, and ^13^C of 100.61 MHz, respectively), equipped with a 4 mm dual broadband CP/MAS probe. The pulp/paper samples were swollen in deionized water overnight before measurement. ^13^C spectra were acquired by using the TOSS (total sideband suppression) sequence at ambient temperature with a spinning rate of 5 kHz, a cross-polarization (CP) a contact time of 2 ms, a recycle delay of 2 s, SPINAL 64 ^1^H decoupling and an acquisition time of 43 ms. Chemical shifts were referenced externally against the carbonyl signal of glycine with *δ* = 176.03 ppm. The acquired FIDs were apodized with an exponential function (lb = 1 Hz) before Fourier transformation. Peak fitting was performed with the Dmfit program. The spectral deconvolution and assignment to cellulose subspecies was performed by spectral fitting according to the model and method of Larsson et al. (1997).^7^

### GPC analysis of cellulose samples

The used protocol is based on Potthast et al. (2015). Samples were characterized by means of the weight-averaged molecular mass (M_w_) obtained from the molecular mass distribution. The cellulosic pulp and paper samples were dissolved in *N,N*-dimethylacetamide containing 9% of lithium chloride (w/v), for the solvent system see Chrapava et al. (2003). The measurement was performed on the GPC system with fluorescence detector (TSP FL2000), multiple-angle laser light scattering detector (Wyatt Dawn DSP with argon ion laser (*λ*_0_ = 488 nm)] and refractive index detector (Shodex RI-71). Separation was performed on a set of four consecutive PLgel mixed-ALS columns (20 µm, 7.5 × 300 mm, Varian/Agilent). *N,N*-Dimethylacetamide containing 0.9% lithium chloride (w/v) was used for mobile phase. The system was operated at a flow rate of 1.0 ml/min with an injection volume of 100 *µ*L. Data evaluation was performed with standard Chromeleon 4, Astra 4.73, and GRAMS/32 software packages.

## Results and discussion

1. Acidic methanolysis

**Fig. 2:**
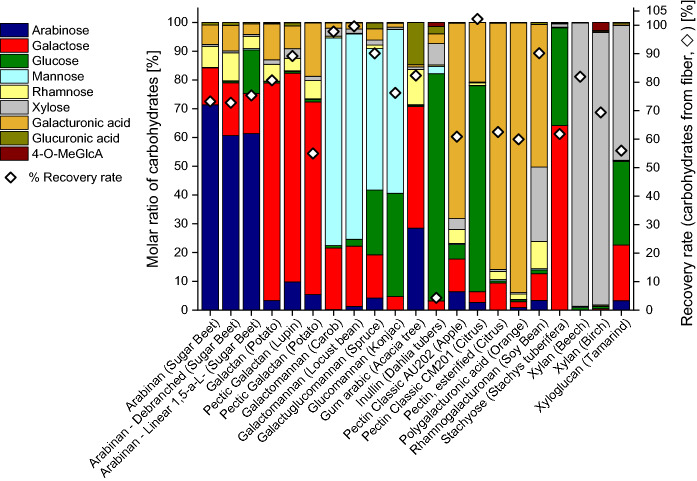
Carbohydrate composition of different polysaccharides, obtained by acidic methanolysis and GC-FID or GC–MS analysis. Molar ratio in % of the total sugar amounts (columns) and recovery rate of released carbohydrates in % (). For more information about the polysaccharide samples see Table 1

Acidic methanolysis proceeded neatly with all tested polysaccharides (Fig. 2) and gave recoveries between 55% and 102.2% of released sugar units, with an average of 83%, which can be regarded as satisfactory. From a conservation science perspective, this is an important result as it confirms that hemicelluloses and auxiliaries often used in conservation treatments can be detected and reliably reported by the method. Only inulin, although completely consumed and solubilized, gave a very poor recovery rate of released carbohydrates of 4.3%. Inulin consists of β-1→2-linked fructofuranose units with a terminal α-1→2 linked glucose unit. While fructose is degraded to furanoid dehydration products during methanolysis, the terminal glucose units are enriched in the mixture and only these are reported.

The primary monosaccharides identified with methanolysis in cellulose samples were glucose, xylose, and mannose, followed by arabinose and galactose (Fig. 2). While glucose originates mainly from cellulose, all other monosaccharides come from hemicelluloses contained (pulps) and/or added auxiliaries (paper). Hydrolysis of these sources (hemicelluloses and additives) was complete as was demonstrated by prolonged reaction times which did not further increase the yield of the contained monosaccharide constituents. However, as expected, the overall recovery rates of released carbohydrates for cellulose-based samples were much lower than in the case of the non-cellulose polysaccharide samples, because a major part of the cellulose materials, mainly the crystalline and other “recalcitrant” regions, are not hydrolyzed (see above).

2. Sulfuric acid hydrolysis (“total hydrolysis”)

**Fig. 3:**
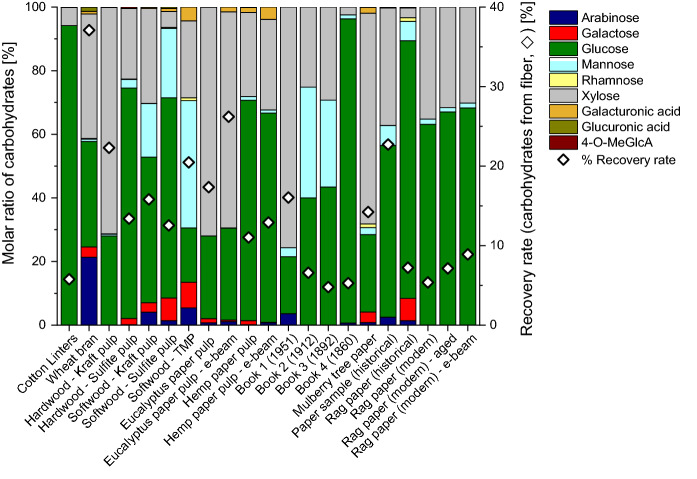
Carbohydrate composition of cellulosic pulp and paper samples, obtained by acidic methanolysis and GC-FID or GC–MS analysis. Molar ratio in % of the total sugar amount (columns) and recovery rate of released carbohydrates in % (). For more information about the cellulose samples see Tables 2 and 3

**Fig. 4:**
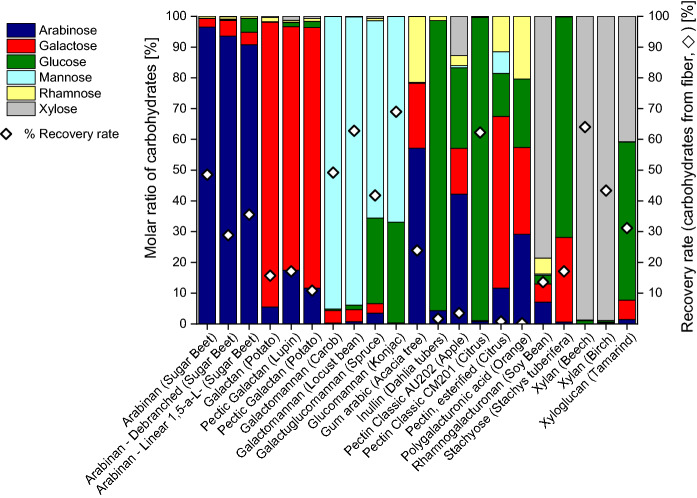
Carbohydrate composition of polysaccharide samples, obtained by sulfuric acid hydrolysis (total hydrolysis) and GC–MS analysis. Molar ratio in % of the total sugar amount (columns) and recovery rate of released carbohydrates in % (). For further information about the polysaccharide samples see Table 1

Sulfuric acid hydrolysis of the polysaccharide samples provided the monosaccharide patterns shown in Fig. 4. A comparison with the data from methanolysis (Fig. 2) showed the main components to be emphasized with values around 90%, *cf.* for instance the high arabinose values in arabinans or the high galactose values in galactans. While also methanolysis reported these monosaccharides to be the main constituents (contents between 60 and 70%), it also showed diverse minor constituents in the single-digit percentage range, such as rhamnose, galactose, glucose and galacturonic acid for arabinans or arabinose, rhamnose, glucose and galacturonic acid for galactans. Sulfuric acid hydrolysis evidently suppressed those minor components. The recovery values of released monosaccharides after sulfuric acid hydrolysis ranged from 0.22 to 69%, with an average of 39%, which was only roughly half of the methanolysis value (83%). The highest recovery values were seen for galactomannan, glucomannan, and xylan, which corresponded to the methanolysis results. The lower recovery and the suppression of minor components are due to the harsh hydrolysis conditions which entail high amounts and large numbers of byproducts, in particular furanoid condensation/dehydration products. Note that sulfuric acid hydrolysis had been introduced for cellulosic materials where the high acidity and the harsh environment is necessary to bring also the recalcitrant higher-order domains to reaction. But these conditions were obviously not optimal for the more labile non-cellulosic polysaccharides studied as the complete solubilization was achieved at the expense of increased byproduct formation and loss of information. This was especially evident in the case of uronic acids: none of the five calibrated uronic acids (galacturonic, glucuronic, 4-*O*-methyl-glucuronic, mannuronic and guluronic acids, the latter two being contained in alginates but not covered in the present study) was observed after sulfuric acid hydrolysis of polysaccharides, not even in traces (Fig. 4), while they were reliably reported by methanolysis (Figs. 2 and 3). These sugar acids undergo decarboxylation and subsequent degradation under the strongly acidic and harsh conditions of sulfuric acid treatment.

A drastic example in this regard was polygalacturonic acid, a pectin: the galacturonic acid content of 56.8% (methanolysis) shrank to zero according to sulfuric acid hydrolysis. Similarly, gum Arabic with 14% of 4-*O*-methyl-glucuronic acid (methanolysis) appeared to contain none of this sugar acid according to sulfuric acid hydrolysis. This virtually forces a cautionary remark from the viewpoint of cellulose conservation: gum Arabic and some pectins have been traditionally used for paper stabilization and conservation treatments. If such papers are examined according to the sulfuric acid hydrolysis method, the presence of such auxiliaries is simply concealed, and it is likely that false conclusions will be drawn about the provenance of the paper, previous conservation treatments and future measures. For such studies, one must resort to the methanolysis method. The focus of interest in such cases is obviously on the additives and on minor components, which are monitored correctly by methanolysis, but not on the—rather obvious—cellulose/glucose content, which would be better measured by total hydrolysis.

**Fig. 5:**
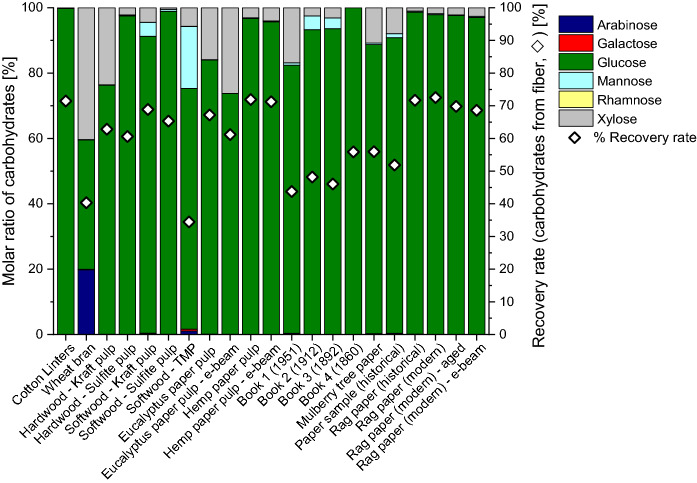
Carbohydrate composition of cellulose samples, expressed as molar ratio in % of the total sugar amount (columns) and recovery rate of released carbohydrates in % () by sulfuric acid hydrolysis and GC–MS analysis. Further information about the samples is shown in Tables 2 and 3

The sulfuric acid hydrolysis (total hydrolysis) of different cellulose samples (Fig. 5) showed higher recovery rates compared to acidic methanolysis (Fig. 3) because the sulfuric acid hydrolysis affected not only the amorphous cellulose fraction but also the crystalline fraction, and also compared to the sulfuric acid hydrolysis of the polysaccharides (Fig. 4) because the largest product component—glucose from cellulose—was more resistant to side reactions and byproduct formation than many of the other monosaccharide units. Recovery rates ranged between 51.9 and 72.5%, except softwood thermomechanical pulp (34.5%) and wheat bran (40.3%), see Fig. 5. The molar ratios of glucose were high and ranged from 39.6 to 100%, followed by xylose (up to 40.3%), and mannose (up to 18.9%). In some samples rhamnose and galactose were reported to be present in concentrations below one percent. Arabinose and uronic acids were not found.

This “simplicity” of the monosaccharide profiles was in complete agreement with the results from polysaccharide total hydrolysis (Fig. 4): several components were suppressed (more correctly: not detected as they were not present due to side reactions) so that the composition appeared less diverse than it actually was (Fig. 3). Although the predominance of glucose in cellulosic substrates is not surprising, the failure to report many minor components from hemicelluloses and pectins is certainly a major drawback of the method. Again, especially the uronic acids were affected, and the destruction of low-content carbohydrates led to a loss of information compared to acidic methanolysis (cf. Figure 3). Incomplete hydrolysis and presence of oligomers was excluded because no signals of disaccharides and trisaccharides were present in GC, and no higher oligomers were detected by HPTLC. The fractions of lipophilic extractives, proteins, and (residual) lignin, and inorganic components were not considered in this study, which influences the recovery values negatively, albeit only to a small extent in the low single-digit percent range.

The acid concentrations, reaction temperatures and processing times of the two-step sulfuric acid hydrolysis method applied in this study was optimized according to Bose et al. (2009) and were adapted for GC/MS analysis. Cellulose crystallinity, the ill-defined term of hornification and the lignin composition of cellulosic samples influence the hydrolysis rates in this procedure (Wijaya et al. 2014) and thus indirectly also the rate and prominence of side reactions. The presence of lignin and condensation/dehydration products from hemicellulose on the surface of cellulose crystallites can negatively affect the cellulose hydrolysis rate (Bhandari et al. 1984; Singh et al. 1984; Zhang et al. 2007). Although the conditions for the two-step sulfuric acid hydrolysis used are generally less harsh than other commonly applied hydrolysis conditions (Bose et al. 2009), the longer reaction times needed to completely hydrolyze such recalcitrant substrates mean at the same time strongly increased degradation of already liberated monosaccharides (Girisuta et al. 2007; Morales et al. 2014).

3. Application of the hydrolysis methods to e-beaming and artificial aging

β-Irradiation (e-beam) treatment is generally used to modify chemical and physical properties of cellulose-containing materials (Henniges et al. 2012, 2013; Driscoll et al. 2009; Sarosi et al. 2020). It has been proposed as pre-treatment of renewable lignocellulosic resources to improve monosaccharide yield for bioethanol production (Postek et al. 2018; Chung et al. 2012; Sundar et al. 2014) or nanocellulose production (Kim et al. 2016; Leskinen et al. 2017; Eo et al. 2016). It was also used as conservation treatment and to clean stained or microbially infested papers (Chosdu et al. 1993; Chmielewska-Śmietanko 2018; Driscoll et al. 2009) and to make papers amenable to reinforcement by synthetic polymer grafts (Kumar and Tumu 2019; Driscoll et al. 2009). Both applications made it especially interesting from the viewpoint of paper conservation in the field of preservation of cultural heritage. The reduction of crystallinity by e-beam radiation has been shown to become evident at doses above 100 kGy (Driscoll et al. 2009; Chung et al. 2012). In our study, paper pulps (eucalyptus and hemp) and rag papers were exposed to e-beam irradiation at 60 kGy or 120 kGy (see Table 2) to find out whether the treatment would have any effects on the results of the two hydrolysis methods.

**Fig. 6:**
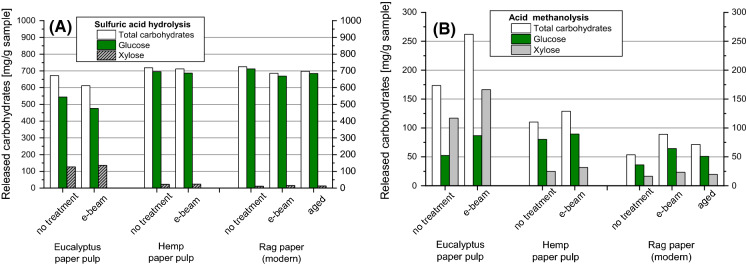
Released carbohydrates (total amount, glucose and xylose) of untreated, β-irradiated and artificially aged cellulose samples. Conditions of e-beam treatment: 120 kGy for eucalyptus and hemp paper pulps, 60 kGy for rag paper. Left (**a**): sulfuric acid hydrolysis (total hydrolysis); right (**b**): methanolysis

The sulfuric acid hydrolysis of e-beam treated pulps indicated a minor loss of carbohydrate yield (− 3.0% for eucalyptus, − 0.9% for hemp and about − 2.0% for rag papers), see Fig. 6. The glucose yield stayed nearly constant (except a yield loss of 4.2% in eucalyptus), and also the xylose yield remained largely unchanged. The outcome was drastically different for the acidic methanolysis, however. Methanolysis reported an increase of the total carbohydrate yield by 51.3% for eucalyptus pulp, 16.9% for hemp pulp, and 66.0% for the rag paper. These differences were indeed massive and much larger than expected. Both the glucose yield and the xylose yield increased significantly (glucose: + 64.9%, + 11.3%, + 77.9% and xylose + 42.2%, + 26.9%, + 45.4% for eucalyptus pulp, hemp pulp and rag paper, respectively), see Fig. 6. Obviously, the e-beam treatment made the materials more accessible to hydrolysis, i.e. it decreased the fraction of reluctant, hard-to-hydrolyze, crystalline fractions. This effect was well reported by the acidic methanolysis method, reflected by a significant gain in carbohydrate yield, which was mainly caused by the gain in glucose yield that came from the cellulose regions now made accessible. In the sulfuric acid system, the yield gain was overcompensated by side reactions, which became even more dominant when more easily hydrolyzable carbohydrate material was present, so that the overall yield stayed constant or even decreased. This result should be kept in mind for studies of the effects of irradiation on cellulosics. An evaluation solely based on total hydrolysis data would provide a largely faulty picture.

Solid-state ^13^C NMR provided only weak support for significant structural changes brought about by β-irradiation. Evaluated by deconvolution of the ordered (86–92 ppm) and less ordered (80–86 ppm) C4-region (Maunu et al. 2000; Nocanda et al. 2007; Zuckerstätter et al. 2009), the percentages of cellulose Iα, cellulose Iβ, “paracrystalline” cellulose, accessible and inaccessible fibril surface and hemicellulose changed less than 2%, see the Supporting Information. It is understandable that the molecular effects of e-beam treatment—mainly radical processes causing chain cleavage and oxidation of near-surface regions with oxygen access—manifest themselves upon swelling, dissolution or chemical modification, but not through such large changes in the largely immobile solid-state structure that they would be detectable by NMR.

**Fig. 7:**
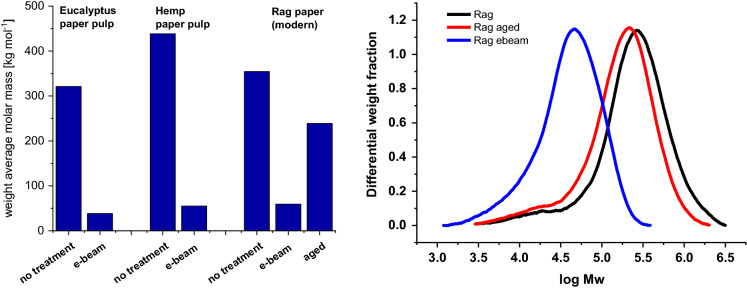
Left: Effect of different treatments (electron beam irradiated (e-beam) and artificially aged cellulose samples (eucalyptus and hemp paper pulps: 120 kGy, and rag paper: 60 kGy) on weight-average molar mass. Right: Molar mass distribution of treated rag papers

In contrast to solid-state NMR, gel permeation chromatography was able to clearly demonstrate the effect of the β-irradiation. A rather pronounced chain cleavage was seen with a significant drop of the weight-averaged molecular mass in the e-beam treated samples (eucalyptus paper pulp: − 88.1%; hemp paper pulp: − 87.4%; rag paper: − 83.3%), see Fig. 7. This is in agreement with the literature which showed similar Mw-loss effects (Saeman et al. 1952; Henniges et al. 2011, 2012, 2013; Hwang et al. 2021).

Accelerated aging, often also called “artificial” aging, is a frequently used approach to simulate natural ageing (Zou et al. 1996; Kato et al. 1999; Ali et al. 2001; Zervos 2010) and to predict the long-term efficiency of conservation treatments. The ageing process was reported to induce structural changes within the paper samples, in particular to change the crystallinity of cellulose. Contradictory opinions are found in the literature with regard to the direction of the crystallinity change in thermally-aged celluloses, with both decrease and increase having been reported (Henniges et al. 2011; Sandy et al. 2010). The impact of environmental parameters on the ageing of paper samples, such as temperature, pressure, air moisture content, presence of oxygen or UV irradiation, has been studied (Baranski 2002; Zou et al. 1996) and degradation kinetic models for artificial ageing processes of papers have been proposed (Emsley and Stevens 1994; Selli et al. 1998, Calvini and Gorassini 2006, Calvini et al. 2008; Kacik et al. 2008). These external parameters, in combination with paper-dependent, internal factors, such as acidity, water content or additive contents (e.g. alum or calcium carbonate), are the key factors influencing the rate of the aging process which, often described by the general term “paper degradation”, is mostly analytically evaluated by the shortening of the cellulose chains, e.g. an Mw loss, or by the accelerated formation of chromophores, e.g. pronounced yellowing, brightness reversion (Korntner et al. 2015; Rosenau et al. 2004) or oxidized functional groups (Ahn et al. 2019; Potthast et al. 2005).

When looking at artificial aging from the perspective of hydrolysis analysis and monosaccharide composition, some similarities can be stated with the β-irradiation treatment. Upon accelerated aging, the total carbohydrate yield of rag paper slightly decreased by sulfuric acid (total) hydrolysis (− 3.7%), but significantly increased according to the acid-catalyzed methanolysis approach (+ 33.2%), as shown in Fig. 6. With regard to glucose recovery, the values were -3.9% for total hydrolysis and + 40.6% for methanolysis. It was evident that the aging procedure increased the accessibility upon hydrolysis, and since this increase was parallel to the increased hydrolytic availability of glucose it must originate from a better access to and destruction of the previously recalcitrant crystalline regions. This was supported by the GPC results: artificial ageing effected an Mw loss in the rag paper of − 32.6%, which is very significant, although not as severe as in the case of e-beam treatment (Fig. 7). This is understandable as the energy input during β-irradiation was much higher, and the subsequent effects, such as radical processes and chain cleavage, consequently more pronounced.

It should be mentioned that our results are somewhat contradictory to those by Sandy et al. (2010) who observed an increase of the crystalline cellulose fraction after ageing experiments, which has later been generalized (Menart et al. 2011). The aging procedure used involved a hydrochloric acid pre-treatment of the paper samples prior to the actual ageing process. It is true that treatment of celluloses with gaseous HCl can increase the crystallinity (Kontturi et al. 2016), but a treatment with aqueous HCl has the opposite effect. The acid pre-treatment followed by the thermal aging generates conditions similar to acid-catalyzed methanolysis. Accessible parts are so severely degraded that they are not recognized as amorphous cellulose and hemicellulose anymore, which results in an apparent increase of crystallinity. This becomes obvious when the monosaccharide yield and GPC data are evaluated as in our study.

## Conclusions

The two hydrolysis methods studied, total hydrolysis with sulfuric acid and methanolysis represent a useful analytical tool for determination of the monosaccharide composition of polysaccharide and cellulose samples in combination with structural changes of the material. The released monosaccharide compounds are quantified by gas chromatographic analysis (GC–MS and GC-FID). Especially the combination of both approaches proved to be useful.

Sulfuric acid hydrolysis is the one of the two methods that completely dissolves and consumes the sample material and also captures the crystalline regions of cellulose, therefore also the common denomination “total hydrolysis”. However, to achieve this full conversion requires rather harsh conditions to be used, so that it comes at the expense of side reactions and degradation of already hydrolyzed/solubilized material. In heterogeneous materials, easily hydrolyzed polysaccharides and readily accessible domains will react fast and soon release their monosaccharides into the harsh medium, where they may undergo side reactions and degradation, while the hard-to-hydrolyze regions may not yet have reacted at all. This becomes a severe problem if non-cellulosic polysaccharides are studied. In particular, glucuronic acids are degraded and not found in the product mixtures, the same is true for ketoses and oxidized monosaccharides with additional carbonyl groups.

Methanolysis as the hydrolysis method behaves in the opposite way. It applies quite mild conditions so that side reactions and degradation of release monosaccharides hardly occur, but it does not hydrolyze the ill-accessible, crystalline regions of cellulose. All monosaccharides are converted into their methyl glucosides, and isomerization causes the appearance of up to four peaks (α/β-furanoses and α/β-pyranoses). Although this increases chromatographic crowding for GC separations, it does not actually mean a drawback, since the risk of complete co-elution is lowered and, as the peak ratio between the isomers is constant for a monosaccharide, quantification can be based on and controlled by more than one peak. As a big plus, side reactions and degradation are minimal. Also uronic acids, which are converted in situ to their methyl esters, can be reliably monitored. Thus, both methods have their pros and cons, and their use must be selected according to the particular sample or analytical problem.

With regard to cellulose conservation science, acid-catalyzed methanolysis was clearly the more valuable of the two approaches. The presence of commonly applied auxiliaries on (historic) paper samples, such as Gum Arabic or pectins, with their high uronate contents, is truthfully reported by methanolysis, but not at all by sulfuric acid hydrolysis which is unsuitable for uronic acid detection. Apart from the conservation aspect, this deficiency of sulfuric acid hydrolysis might also become important if the method is applied to TEMPO-oxidized cellulosic materials, such as the frequently utilized TEMPO-oxidized cellulose nanofibrils or polyglucuronic acids, or alginates (mannuronic and guluronic acids). In these cases, only methanolysis can be used for monosaccharide analysis as the sulfuric acid alternative fails to capture uronic acids and gives grossly erroneous results. Similarly, the minor contents of oxidized groups (carbonyl and carboxyl) in oxidatively damaged cellulosic would never by correctly reported by the total hydrolysis method.

Also with respect to paper conservation treatments, such as β-irradiation (e-beam treatment) and accelerating aging, acid-catalyzed methanolysis was superior to its H_2_SO_4_ counterpart. The latter indirectly showed a decrease in crystallinity, for both methods, but especially for β-irradiation, because of a faster hydrolysis reaction and a decrease of the total monosaccharide yield due to more side reactions. Methanolysis, on the other hand, showed a significantly boosted monosaccharide yield (mainly due to a gain of glucose from cellulose). These results were independent of the sample type. It became clear that both treatments significantly increased the accessibility and the reactivity upon hydrolysis. This is accompanied (or caused) by a drastic loss of molecular weight as seen by GPC. GPC was well able to report the changes caused by the treatments, as was methanolysis, whereas solid-state NMR showed very little spectral change.

The β-irradiation treatment improved carbohydrate yield and polysaccharide accessibility in cidic hydrolysis—probably this is equally true for enzyme treatments. This makes the method interesting as pretreatment option for biomass feedstocks in biofuel conversion. In conservation science, the intended benefits, e.g. sterilization or elimination of pest infestation, must be balanced with the obvious negative effect of the structural changes and Mw loss. The effect of accelerated aging was clearly seen by the methanolysis approach—and fully agreed with GPC results. However, it remains to be seen whether these effects are the same as those seen in natural aging of paper materials, and whether the accelerated aging is thus a suitable mimic of the natural process.

## Supplementary Information

Below is the link to the electronic supplementary material.

Supplementary file1 (DOCX 2769 kb)
