# Supplementary material for: Comparative hydrolysis analysis of cellulose samples and aspects of its application in conservation science
Source: Cellulose (Lond). 2021 Jul 23;28(13):8719–34. doi: 10.1007/s10570-021-04048-6 (PMC8299441; doi:10.1007/s10570-021-04048-6)
Supplement: Supplementary file 1 — Supplementary file1 (DOCX 2769 kb) [file 10570_2021_4048_MOESM1_ESM.docx]

**Supporting information:****Comparative hydrolysis analysis of cellulose samples and aspects of its application in conservation science**

Manuel Becker^a^, Kyujin Ahn^a,c^, Markus Bacher ^a^, Chunlin Xu^b^, Anna Sundberg^b^, Stefan Willför^b^, Thomas Rosenau^a,b^, Antje Potthast^a^*

^a^ Department of Chemistry, Institute of Chemistry of Renewables University of Natural Resources and Life Sciences, Muthgasse 18, A-1190 Vienna, Austria

^b^ Johan Gadolin Process Chemistry Centre, c/o Laboratory of Natural Materials Technology, Åbo Akademi University, Porthaninkatu 3, 20500 Turku, Finland

^c^ National Archives of Korea, 30 Daewangpangyo-ro 851beon-gil, Sujeong-gu, Seongnam-si, Korea

* Corresponding author, antje.potthast@boku.ac.at

Content:

Table S1: Crystallinity index of cellulose samples from CPMAS

Figure S1: Signals of spectral fitting of the cellulose C4-region obtained from ^13^C CP-MAS NMR spectra of eucalyptus and hemp paper pulps and rag paper (from above to bottom), before (left) and after electronic beam (e-beam) treatment (right). The red line shows the experimental spectra. The colorized solid lines represent the deconvoluted signals/fractions: I_α_ and I_β_ – Crystalline cellulose I_α_ and I_β_; Para – Paracrystalline cellulose; AFS – Accessible fibril surfaces; IAFS – Inaccessible fibril surfaces; Hemi – Hemicellulose.

***Solid state NMR data.***

The spectral fitting results of the NMR C4-region spectra obtained from eucalyptus and hemp paper pulps and rag paper before and after e-beam treatment are shown in Figure S1.

Figure S1: Signals of spectral fitting of the cellulose C4-region obtained from ^13^C CP-MAS NMR spectra of eucalyptus and hemp paper pulps and rag paper (from above to bottom), before (left) and after electronic beam (e-beam) treatment (right). The red line shows the experimental spectra. The colorized solid lines represent the deconvoluted signals/fractions: I_α_ and I_β_ – Crystalline cellulose I_α_ and I_β_; Para – Paracrystalline cellulose; AFS – Accessible fibril surfaces; IAFS – Inaccessible fibril surfaces; Hemi – Hemicellulose.

Table S1: Crystallinity index of cellulose samples from CPMAS

| **Sample** | **CRI** |
| --- | --- |
| Wheat bran | 4.17 |
| TMP Spruce | 20.86 |
| Paper sample (1912, F13) | 32.87 |
| Birch Sulfat | 38.32 |
| Avicel | 42.04 |
| Mulberry tree paper | 44.33 |
| Beech sulfite pulp | 48.63 |
| Encell | 49.16 |
| Softwood Kraft | 53.97 |
| Softwood - Sulfite | 56.47 |
| Hempcell | 61.49 |
| Rag paper (historical) | 64.48 |
| Cotton linters | 65.78 |
| Rag paper (modern) | 67.61 |
|  |  |

Detailed data from CPMAS

**Cotton linters**

|  | δ [ppm] | width [ppm] | integral / % |
| --- | --- | --- | --- |
| I_α_ | 89.56 | 0.41 | 2.78 |
| I_α+β_ | 88.81 | 0.43 | 16.64 |
| I_β_ | 87.98 | 0.70 | 13.87 |
| paracrystalline | 88.43 | 1.81 | 27.57 |
| accessible fibril surface | 84.21 | 1.20 | 6.12 |
| accessible fibril surface | 83.22 | 0.74 | 3.45 |
| inaccessible fibril surface | 83.37 | 7.93 | 28.30 |
| hemicellulose | 82.27 | 0.96 | 1.28 |

crystallinity index:

manual integration: crystalline : amorphous = 1 : 0.47

peak fitting result: crystalline : amorphous = 1 : 0.64 (with hemicellulose)

crystalline : amorphous = 1 : 0.62 (without hemicellulose)

**Hempcell**

|  | δ [ppm] | width [ppm] | integral / % |
| --- | --- | --- | --- |
| I_α_ | 89.54 | 0.63 | 4.10 |
| I_α+β_ | 88.79 | 0.53 | 19.35 |
| I_β_ | 87.97 | 0.91 | 21.78 |
| paracrystalline | 88.48 | 1.91 | 14.01 |
| accessible fibril surface | 84.25 | 0.95 | 3.50 |
| accessible fibril surface | 83.23 | 0.77 | 2.77 |
| inaccessible fibril surface | 82.93 | 5.86 | 33.15 |
| hemicellulose | 81.93 | 0.98 | 1.34 |

crystallinity index:

manual integration: crystalline : amorphous = 1 : 0.64

peak fitting result: crystalline : amorphous = 1 : 0.69 (with hemicellulose)

crystalline : amorphous = 1 : 0.67 (without hemicellulose)

**Encell**

|  | δ [ppm] | width [ppm] | integral / % |
| --- | --- | --- | --- |
| I_α_ | 89.50 | 0.71 | 3.26 |
| I_α+β_ | 88.80 | 0.56 | 7.28 |
| I_β_ | 87.86 | 0.84 | 2.68 |
| paracrystalline | 88.40 | 1.92 | 26.15 |
| accessible fibril surface | 84.27 | 1.19 | 5.19 |
| accessible fibril surface | 83.22 | 0.69 | 2.90 |
| inaccessible fibril surface | 83.17 | 6.72 | 42.93 |
| hemicellulose | 81.94 | 1.74 | 9.61 |

crystallinity index:

manual integration: crystalline : amorphous = 1 : 1.20

peak fitting result: crystalline : amorphous = 1 : 1.54 (with hemicellulose)

crystalline : amorphous = 1 : 1.30 (without hemicellulose)

**Birch sulfate**

|  | δ [ppm] | width [ppm] | integral / % |
| --- | --- | --- | --- |
| I_α_ | 89.51 | 0.71 | 2.43 |
| I_α+β_ | 88.82 | 0.56 | 4.30 |
| I_β_ | 87.72 | 0.72 | 1.02 |
| paracrystalline | 88.49 | 2.03 | 27.03 |
| accessible fibril surface | 84.22 | 1.31 | 5.05 |
| accessible fibril surface | 83.20 | 0.76 | 3.15 |
| inaccessible fibril surface | 82.96 | 6.77 | 14.92 |
| hemicellulose | 81.90 | 1.89 | 42.09 |

crystallinity index:

manual integration: crystalline : amorphous = 1 : 1.47

peak fitting result: crystalline : amorphous = 1 : 1.87 (with hemicellulose)

crystalline : amorphous = 1 : 0.66 (without hemicellulose)

**modern rag**

|  | δ [ppm] | width [ppm] | integral / % |
| --- | --- | --- | --- |
| I_α_ | 89.58 | 0.54 | 2.93 |
| I_α+β_ | 88.83 | 0.46 | 14.88 |
| I_β_ | 87.99 | 0.75 | 13.67 |
| paracrystalline | 88.49 | 1.79 | 20.53 |
| accessible fibril surface | 84.25 | 1.15 | 5.98 |
| accessible fibril surface | 83.24 | 0.76 | 3.53 |
| inaccessible fibril surface | 82.79 | 9.18 | 35.44 |
| hemicellulose | 82.09 | 1.30 | 3.04 |

crystallinity index:

manual integration: crystalline : amorphous = 1 : 0.65

peak fitting result: crystalline : amorphous = 1 : 0.92 (with hemicellulose)

crystalline : amorphous = 1 : 0.86 (without hemicellulose)

**historical rag**

|  | δ [ppm] | width [ppm] | integral / % |
| --- | --- | --- | --- |
| I_α_ | 89.59 | 0.52 | 2.70 |
| I_α+β_ | 88.84 | 0.49 | 14.36 |
| I_β_ | 88.00 | 0.71 | 10.09 |
| paracrystalline | 88.49 | 1.89 | 27.54 |
| accessible fibril surface | 84.29 | 1.12 | 6.64 |
| accessible fibril surface | 83.26 | 0.87 | 4.42 |
| inaccessible fibril surface | 83.45 | 8.35 | 33.06 |
| hemicellulose | 82.20 | 1.06 | 1.19 |

crystallinity index:

manual integration: crystalline : amorphous = 1 : 0.58

peak fitting result: crystalline : amorphous = 1 : 0.81 (with hemicellulose)

crystalline : amorphous = 1 : 0.78 (without hemicellulose)

**historical book paper 1912 F13**

|  | δ [ppm] | width [ppm] | integral / % |
| --- | --- | --- | --- |
| I_α_ | 89.54 | 0.56 | 2.34 |
| I_α+β_ | 88.82 | 0.54 | 8.39 |
| I_β_ | 87.98 | 0.80 | 4.30 |
| paracrystalline | 88.55 | 2.01 | 29.29 |
| accessible fibril surface | 84.25 | 1.20 | 7.32 |
| accessible fibril surface | 83.24 | 0.79 | 3.89 |
| inaccessible fibril surface | 83.18 | 6.95 | 39.99 |
| hemicellulose | 81.94 | 1.59 | 4.47 |

crystallinity index:

manual integration: crystalline : amorphous = 1 : 0.97

peak fitting result: crystalline : amorphous = 1 : 1.26 (with hemicellulose)

crystalline : amorphous = 1 : 1.16 (without hemicellulose)

**Beech sulfite pulp**

|  | δ [ppm] | width [ppm] | integral / % |
| --- | --- | --- | --- |
| I_α_ | 89.51 | 0.55 | 1.68 |
| I_α+β_ | 88.81 | 0.54 | 8.27 |
| I_β_ | 87.80 | 0.66 | 1.55 |
| paracrystalline | 88.49 | 1.86 | 29.81 |
| accessible fibril surface | 84.28 | 1.14 | 10.01 |
| accessible fibril surface | 83.24 | 0.80 | 6.00 |
| inaccessible fibril surface | 83.88 | 7.30 | 40.24 |
| hemicellulose | 82.08 | 1.14 | 2.44 |

crystallinity index:

manual integration: crystalline : amorphous = 1 : 0.97

peak fitting result: crystalline : amorphous = 1 : 1.42 (with hemicellulose)

crystalline : amorphous = 1 : 1.36 (without hemicellulose)

**softwood kraft pulp**

|  | δ [ppm] | width [ppm] | integral / % |
| --- | --- | --- | --- |
| I_α_ | 89.57 | 0.23 | 0.39 |
| I_α+β_ | 88.79 | 0.57 | 10.17 |
| I_β_ | 87.87 | 1.09 | 9.69 |
| paracrystalline | 88.61 | 2.05 | 26.94 |
| accessible fibril surface | 84.25 | 0.98 | 5.65 |
| accessible fibril surface | 83.22 | 0.69 | 3.66 |
| inaccessible fibril surface | 82.74 | 6.39 | 42.17 |
| hemicellulose | 81.84 | 1.07 | 1.34 |

crystallinity index:

manual integration: crystalline : amorphous = 1 : 1

peak fitting result: crystalline : amorphous = 1 : 1.12 (with hemicellulose)

crystalline : amorphous = 1 : 1.09 (without hemicellulose)

**softwood sulfite pulp**

|  | δ [ppm] | width [ppm] | integral / % |
| --- | --- | --- | --- |
| I_α_ | 89.57 | 0.43 | 1.21 |
| I_α+β_ | 88.81 | 0.49 | 6.98 |
| I_β_ | 87.67 | 1.14 | 4.73 |
| paracrystalline | 88.58 | 1.82 | 34.33 |
| accessible fibril surface | 84.28 | 1.03 | 7.48 |
| accessible fibril surface | 83.28 | 0.85 | 6.34 |
| inaccessible fibril surface | 83.85 | 7.10 | 38.07 |
| hemicellulose | 82.18 | 0.93 | 0.85 |

crystallinity index:

manual integration: crystalline : amorphous = 1 : 0.78

peak fitting result: crystalline : amorphous = 1 : 1.12 (with hemicellulose)

crystalline : amorphous = 1 : 1.10 (without hemicellulose)

**Mulberry paper MB (Hanji paper H)**

|  | δ [ppm] | width [ppm] | integral / % |
| --- | --- | --- | --- |
| I_α_ | 89.60 | 0.63 | 3.00 |
| I_α+β_ | 88.82 | 0.49 | 14.15 |
| I_β_ | 87.99 | 0.80 | 11.92 |
| paracrystalline | 88.48 | 1.78 | 20.94 |
| accessible fibril surface | 84.24 | 1.24 | 7.21 |
| accessible fibril surface | 83.26 | 0.77 | 3.61 |
| inaccessible fibril surface | 83.25 | 8.90 | 35.78 |
| hemicellulose | 82.33 | 1.63 | 3.37 |

crystallinity index:

manual integration: crystalline : amorphous = 1 : 0.73

peak fitting result: crystalline : amorphous = 1 : 0.93

**wheat bran - No fitting possible**

**TMP spruce - No fitting possible**

**Hempcell _ e-beam**

|  | δ [ppm] | width [ppm] | integral / % |
| --- | --- | --- | --- |
| I_α_ | 89.51 | 0.76 | 4.09 |
| I_α+β_ | 88.78 | 0.66 | 17.13 |
| I_β_ | 87.97 | 0.97 | 14.07 |
| paracrystalline | 88.29 | 1.91 | 19.49 |
| accessible fibril surface | 84.25 | 0.95 | 3.30 |
| accessible fibril surface | 83.23 | 0.96 | 3.35 |
| inaccessible fibril surface | 83.19 | 6.16 | 36.84 |
| hemicellulose | 81.93 | 0.99 | 1.72 |

crystallinity index:

manual integration: crystalline : amorphous = 1 : 0.71

peak fitting result: crystalline : amorphous = 1 : 0.83 (with hemicellulose)

crystalline : amorphous = 1 : 0.79 (without hemicellulose)

**Rag paper - e-beam**

|  | δ [ppm] | width [ppm] | integral / % |
| --- | --- | --- | --- |
| I_α_ | 89.56 | 0.61 | 2.59 |
| I_α+β_ | 88.82 | 0.54 | 14.05 |
| I_β_ | 87.98 | 0.82 | 12.60 |
| paracrystalline | 88.52 | 1.87 | 19.96 |
| accessible fibril surface | 84.20 | 1.30 | 7.03 |
| accessible fibril surface | 83.22 | 0.76 | 2.78 |
| inaccessible fibril surface | 83.29 | 10.30 | 36.71 |
| hemicellulose | 82.14 | 1.53 | 4.28 |

crystallinity index:

manual integration: crystalline : amorphous = 1 : 0.70

peak fitting result: crystalline : amorphous = 1 : 1.03 (with hemicellulose)

crystalline : amorphous = 1 : 0.95 (without hemicellulose)

**Encell I e-beam**

|  | δ [ppm] | width [ppm] | integral / % |
| --- | --- | --- | --- |
| I_α_ | 89.48 | 0.68 | 2.15 |
| I_α+β_ | 88.78 | 0.62 | 6.64 |
| I_β_ | 88.00 | 1.10 | 3.98 |
| paracrystalline | 88.45 | 2.10 | 25.77 |
| accessible fibril surface | 84.26 | 0.93 | 3.38 |
| accessible fibril surface | 83.23 | 0.81 | 3.07 |
| inaccessible fibril surface | 82.95 | 6.10 | 49.91 |
| hemicellulose | 81.87 | 1.30 | 5.10 |

crystallinity index:

manual integration: crystalline : amorphous = 1 : 1.25

peak fitting result: crystalline : amorphous = 1 : 1.59 (with hemicellulose)

crystalline : amorphous = 1 : 1.46 (without hemicellulose)
